# Some of the Early Experiences of an Old Physician

**Published:** 1898-04

**Authors:** G. G. Roy

**Affiliations:** Atlanta, Ga.


### SOME OF THE EARLY EXPERIENCES OF AN OLD PHYSICIAN.

BY G. G. ROY, M.D.,

ATLANTA, GA.

In the March number of THE JOURNAL I gave a history of my first patient, in 1857. It cannot be so well appreciated by the young physicians of this day as by those who began practice in the South when negroes were the property of their owners, and very valuable property, the care and preservation of which, separate and apart from the standpoint of the humanitarian, was enhanced by the more sordid motive of self-interest. It was losing money to lose a negro by death, and it was saving money to save the life of one when ill. Consequently, in those days the negro when sick received the most skilful medical attention, the very best nursing, and every comfort was added to these that could increase the chances of recovery.

The young physician who was fortunate enough to get the practice of two or three large plantations had a bonanza. Bills then were more easily collected and they were paid more readily and cheerfully than our bills are at this progressive age of American freedom.

Negroes were then superstitious, very; believed in "cunjuration," being "tricked," and in ghosts, hobgoblins, jack-o'-lanterns, etc. Nor are they now, with the millions of dollars expended yearly for their education, in these respects very far removed from those innate conditions of the old Southern slave. Superstition is an instinct or intuition of the negro, and it will not down by education, and it will not desert the race until every vestige of the character of the old-time Southern darky is purged from the world. And when that is done the race will be extinct in the land.

But I began to tell of my second patient when I started out as a full-fledged doctor. I was called to see a man with whom I grew up from a boy. He had developed into an oysterman and fisherman, a very lucrative business in those days. He lived near the

Rappahannock river in tidewater Virginia, noted the world over for its fine oysters and fish—especially the former. Tom was expert at both, and had been very successful, not only in this business, but more so as a dram-drinker. The shores of the river in that section of the State were studded with little groceries, all of which dispensed liquors. This, too, was a profitable business; they were the catch-nets for the money of the oystermen and fishermen. Tom began moderately, and kept this up for several years; finally the habit grew and he became a sot, and by this time he had produced what the doctors know as an alcoholic or whisky liver. The inevitable result soon followed—general dropsy. And this was his condition when I was called to see him. On my road to his house I had to pass the farm of an old friend who had known me from my babyhood. He was sitting on the fence watching, or, as we called it in those days, overseeing his negroes at work in his cornfield. He had not seen me before since I had gotten home with my sheepskin, and of course stopped me for a chat. "Well, Gus," he said, "you have come home a full-fledged doctor, and are going out to kill or cure, the former of which you will be very sure to do. I want to tell you right here and now, you will never make the doctor your father is." This was very galling to my pride and vanity, and I thought he was very impertinent. I certainly was very much disgusted. But he did not stop at this. Said he: "You are going to see Tom McK——. He has the dropsy, and I can cure him now quicker than you can. I have a remedy that never fails." By this time patience had ceased to be a virtue, and, without making a reply or bidding him good-bye, I spurred my horse in the direction of my patient's home.

Reaching it, I found poor Tom swollen until every feature of himself as I knew him was obliterated. Face swollen until his eyes were tightly closed; he could not see me, but recognizing my voice he said, "Is that you, Gus?" (Now, do you know of anything that will make a *right young* doctor madder than to call him by his first name, or Mr.?) But, poor boy, he was so disfigured, and seemed to me to be so nearly dead, I quickly forgave him. His abdomen was swelled till about the size of a keg of beer, his legs and feet looked like those of an elephant. I felt so sorry for Tom, because he had a big heart and was kind to everybody but himself.

I went vigorously to work; read all the books on dropsy in mine and my father's libraries; laid awake at night thinking about his case; would dream of a new remedy I had forgotten; would get up, light the candle, get my books and reread all I had read looking for it, and to find out what the *books* said about it; I would then get my college note-books to see if the professors had not mentioned something about such a case that I could not find in the books.

I began my treatment, visited him every day for a week; but poor Tom did not get any smaller. I was becoming very miserable myself, to say nothing of Tom's feelings. At my next visit I chanced to find my old friend sitting on the same fence, but in another place. He stopped me as before, and said, "Well, Gus, how is Tom getting on?" I summoned all the courage I had (my heart was then in my boots), and said, "Oh, doing very well."

I said to him, "You spoke of having a good remedy—what is it?" Now, he was one of these farmer-doctors (no quack) who took great pleasure in telling his remedies and his successes with them. He said, with a confidence born of self-reliance if not knowledge: "You take two teaspoonfuls of nicely powdered copperas and four tablespoonfuls of Epsom salts; mix them well together; put into a quart bottle and fill it with water nearly hot (don't use boiling water, if you do you will *bust* the bottle). Shake it well every time you give it, and give from one to two tablespoonfuls every three or four hours, till you make his bowels act like lightning. Do this for two or three days, and when the swelling begins to go down give it three times a day, and I'll be darned if you don't have him as thin as a whippoorwill in a week."

My remedies having failed, I accepted that of my farmer-doctor. (But I did not tell him so. This is a point it is well for the young doctor to remember.) I gave him the salts and copperas as directed, and it had just the effect the old farmer claimed for it. In a week's time all of poor Tom's swelling was gone and he was able to sit around, though, as you may readily imagine, *very weak*.

Dropsy is a symptom, not a disease, *per se*. There are three kinds of dropsy ordinarily met with: the liver, heart, and kidney dropsy. The first is most common in abusers of alcoholic drinks and in malarial districts, and is more amenable to treatment than either of the other two. When we have a dropsy from organic disease of the heart or kidneys, you can only give temporary relief. It may not be difficult in either case at the first two or three attacks to reduce the swelling by active drastic purgation, alternated with those medicines which will produce active and profuse diuresis. But in heart and kidney dropsies the effusion quickly returns, and your remedies for its removal will all fail; then you resort to paracentesis, which will relieve the urgent and distressful symptoms for awhile, then they all fail, and death ensues.

Dropsy produced from obstruction of the portal circulation is sometimes permanently relieved, because the condition which produces it is amenable to radical cure by proper medication and attention to diet and drink. But it must be remembered that reducing the swelling is not *curing* the dropsy. This is just what and just all the quack dropsy doctors do—relieve the swelling—and every time they do this they claim a cure, and the next time it is a new bill for a new case. They all depend upon Epsom salts, giving from one-half to one pound a day; but they haven't the sense my farmer friend had of adding the iron, so as to strengthen the blood, and thereby increase nutrition while getting rid of the effusion. This is the secret of curing curable cases of dropsy: begin with some soluble ferruginous preparation just as soon as you see the swelling begin to go down.

The salts and copperas—or, as he called it, his ferro-saline—was a favorite remedy of Professor Robley Dunglison, when he occasionally held clinics at the Jefferson Medical College, forty years ago, and I have used it in various forms in different conditions of general anemia ever since, and have found no better.

(*To be continued.*)

---

A BALTIMORE judge has decided that faith-cure doctors are not legally entitled to remuneration for their services. He takes the ground that the faith-cure physician renders no apparent service to the sick.—*Exchange.*

